# Same‐Day Discharge After Atrial Fibrillation Ablation. A Scoping Review of Nurse‐Led Care Model

**DOI:** 10.1002/hsr2.72127

**Published:** 2026-03-19

**Authors:** Domenico Pascucci, Silvia Martinelli, Mario Cesare Nurchis, Antonello Cocchieri, Francesca Notarangelo, Maria Lucia Narducci, Walter Ricciardi, The Nursing and Public Health Group, Elena Cristofori, Vittorio De Vita, Anna Nisticò, Lia Olivo, Manuele Cesare, Erasmo Magliozzi, Gianfranco Damiani

**Affiliations:** ^1^ Section of Hygiene, Department of Health Sciences and Public Health Università Cattolica del Sacro Cuore Rome Italy; ^2^ Health Management Fondazione Policlinico Universitario A. Gemelli IRCCS Rome Italy; ^3^ Cardiology Department Parma University Hospital Parma Italy; ^4^ Department of Cardiovascular Sciences Fondazione Policlinico Universitario A. Gemelli IRCCS Rome Italy; ^5^ Department of Woman and Child Health and Public Health Fondazione Policlinico Universitario A. Gemelli IRCCS Roma Italy; ^6^ Università Cattolica del Sacro Cuore Rome Italy; ^7^ Center of Excellence for Nursing Scholarship Rome Italy; ^8^ University of Rome Tor Vergata Rome Italy

**Keywords:** atrial fibrillation, atrial fibrillation ablation, nurse‐led care model, nurse‐led health management, same‐day discharge

## Abstract

**Background and Aims:**

Atrial fibrillation (AF) is a common cardiac arrhythmia with a rising global incidence. Advances in ablation techniques have led to same‐day discharge (SDD) protocols, reducing hospital resource use. However, managing patients post‐ablation is crucial to avoid complications. This scoping review evaluates the role of nurse‐led interventions in managing patients undergoing AF ablation with SDD, focusing on patient safety, outcomes, and healthcare efficiency.

**Methods:**

A review was conducted across databases like MEDLINE, Web of Science, Scopus, and Google Scholar in April 2024. Studies were included if they reported on nurse‐led management during hospitalization or post‐ablation follow‐up in adult patients.

**Results:**

Ten clinical studies involving 4776 patients were reviewed. The studies showed that nurse‐led interventions effectively support SDD protocols, ensuring patient safety and improving outcomes. Interventions varied, including hotlines, follow‐up calls, and autonomous discharge decisions by nursing staff. Overall complication rates for SDD ranged from 0% to 18%, with most studies reporting ≤ 1.6%. Minor complications occurred in up to 3.5% of day cases, re‐hospitalization rates reached 2.1%, and unplanned overnight admissions for minor events were up to 7.3%. These rates were generally comparable to, or lower than, those for overnight stay (ONS) procedures.

**Conclusion:**

Nurse‐led models facilitated early complication detection, enhanced patient education, and improved follow‐up care. The reported complication rates for SDD were low and comparable to ONS, supporting its safety, feasibility, and potential to reduce healthcare resource use. Further research should standardize protocols and assess their long‐term impact.

## 1 Introduction

Atrial fibrillation (AF) is one of the most commonly encountered cardiac arrhythmias, with a broad impact on all health services across primary and secondary care. The prevalence of AF is expected to double in the next few decades as a consequence of the ageing population, an increasing burden of comorbidities and improved detection [1, 2, 3, 4, 5, 6, 7]. Particularly, the number of AF cases in the U.S. could reach up to 16 million by 2050, with Europe and Asia expecting similar rises to 14 million by 2060 [2]. The total Disability‐Adjusted Life Years (DALYs) attributed to AF increased from 3,787,838 (95% CI 267,964–361,014) in 1990 to 8,393,635 (95% CI 267,964–361,014) in 2019. However, the age‐standardized DALY rate remained relatively stable between over this period [8].

Patients with AF exhibit significantly higher mortality rates, primarily due to various complications and causes of death [9]. Stroke and bleeding events are also notable complications, each contributing to 7% of all deaths among AF patients [10, 11]. Analysis of national data indicates that the mortality risk from these cardiovascular complications has been rising, particularly among younger adults [12].

AF is imposing significant financial challenges on healthcare systems in the USA and Europe, primarily due to hospitalizations and complications like stroke [13, 14]. In the USA, annual AF treatment costs exceed $6.65 billion, primarily from hospital and outpatient care [13]. European costs range from €660 to €3286 million annually, with similar drivers [14]. Both regions incur high indirect costs from AF‐related complications and managing comorbid conditions. The growing AF patient population is expected to increase healthcare demands and costs, highlighting the urgent need for effective management strategies in both the USA and Europe [14].

With regard to the recent ESC guidelines 2024 [5], the treatment of AF typically begins with the CARE approach (Comorbidity management, Avoid Stroke and thromboembolism, Rate or rhythm control, Evaluation and dynamic assessment). In particular, as rhythm control strategy, catheter ablation (CA) prevents AF recurrences, reduces AF burden, and improves quality of life in symptomatic paroxysmal or persistent AF where the patient is intolerant or does not respond to anti‐arrhythmic drugs [15]. Multiple randomized controlled trials [16, 17] have shown that CA more effectively restores normal sinus rhythm compared to antiarrhythmic drug therapy alone, with a success rate of 71% (95% CI, 65%–77%) when combined with drug therapy, vs. 57% (95% CI, 50%–64%) with drugs alone; CA also results in a lower recurrence rate of AF at 20%, compared to 75% with drug therapy alone [18]. CA is recommended due to its effectiveness in improving the quality of life and ventricular function [19], showing significant benefits after both single and multiple procedures.

As the technical aspects of AF CA have evolved, with reduced procedure times, different types of energy and lower complication rates, same‐day discharge (SDD) for AF ablation has increasingly been implemented to minimize the impact on hospital resources [20], given the growing number of AF CA [21]. A few systematic reviews [22, 23] have explored the safety and efficacy of same‐day discharge following CA for AF (AF). A recent comprehensive review [22], encompassing 14 observational studies with 26,488 patients, revealed that 9766 were discharged the same day with a success rate of 83.2%. The findings indicated no significant difference in 30‐day complications or readmissions between SDD and overnight stay groups supporting the safety and efficiency of SDD in clinical practice, offering a viable option for reducing healthcare utilization without increasing risk. A recent meta‐analysis [24] showed SDD after AF CA was associated with low prevalence of post‐discharge complications, re‐hospitalizations/ER visits and mortality, and a similar risk compared with overnight stay (ONS).

In response to the COVID‐19 pandemic, healthcare systems have increasingly adopted innovative and resource‐efficient care models, with nurse‐led interventions playing a significant role [25, 26, 27]. These interventions are based on a holistic care delivery model that integrates assessment, evaluation, education, counseling, treatment, and other procedures through a comprehensive approach involving both the patient and their family. Nurse‐led interventions can be carried out independently by nursing professionals or as part of interdisciplinary teams. Over time, this patient‐centered, multidisciplinary approach has been implemented across various fields, including oncology, heart failure, diabetes mellitus, and cardiac arrhythmias like AF [28]. Such an approach is particularly critical in managing AF CA, especially as the push for shorter hospital stays intensifies due to resource constraints. The trend toward SDD following CA highlights the importance of effective nurse‐led follow‐up care to improve patient outcomes, manage the immediate post‐procedure period, address variations in recovery, and reduce the risk of complications.

The definition of nurse‐led care emphasizes a structured interaction between nurse and patient and their family, focusing on continuity of care, a critical component of high‐quality healthcare associated with improved preventive care, reduced hospitalization, and patient satisfaction [29, 30]. Despite the growing evidence supporting the nurse's role in this area, gaps remain in linking specific nursing interventions to improved outcomes.

Given the current healthcare demands and the potential for nurse‐led models to deliver cost‐effective, patient‐centered care, understanding and expanding the evidence base for nurse‐led care in SDD settings is crucial. This review aims to explore the existing literature on nurse‐led management in SDD for AF CA, mapping intervention types, assessing their documented effectiveness where possible, and evaluating the potential for practical implementation.

## 2 Methods

### 2.1 Study Design

A scoping review of the literature was conducted to map the evidence on nurse‐led care model in SDD for AF ablation adopting the methodological framework suggested by Arksey and O'Malley [31] and refinements by the Joanna Briggs Institute [32].

The final protocol was registered prospectively with the Open Science Framework on April 30, 2024 [33].

### 2.2 Search Strategies

The population, concept, and context (PCC) framework [34] was adopted to frame the following guiding question of the scoping review: “What evidence exists in the literature regarding the effectiveness of nurse‐led interventions for patients undergoing ablation for atrial fibrillation in a same‐day discharge setting?” “Nurse‐led care” refers to care overseen and coordinated by a nurse with advanced skills, involving assessment, planning, management, and coordination of patient care. This includes promoting continuity of care and integration across various healthcare services and providers. The nurse's role extends beyond autonomous decision‐making to encompass organizing activities in alignment with the overall care goal, ensuring collaboration among multiple involved parties [35, 36, 37]. Initially, the search string was constructed by combining the following keywords: “Atrial fibrillation,” “AF,” “ablation,” “nurse‐led,” “Early discharge,” “Same‐Day procedure,” “Same‐day discharge,” “SDD,” “Outpatient ablation,” “Day Service,” and their synonyms using Boolean operators “AND” and “OR.” However, preliminary testing of the search strategy showed that including “nurse‐led” and equivalent terms substantially reduced the number of retrieved records, potentially missing relevant studies where the nurse‐led component was present but described using different terminology and not using that specific term. For this reason, these terms were removed from the final search string, and the “nurse‐led intervention” criterion was instead applied rigorously during title/abstract and full‐text screening. The complete search strategy is reported in Supplementary 1. The search was completed using the snowball search technique to retrieve additional studies or references from the included articles.

The search string was introduced in PubMed, Scopus, and Web of Science databases, spanning from their inception to March 2024. To ensure a thorough exploration of relevant studies, Google Scholar was also included as a supplementary resource.

The Preferred Reporting Items for Systematic Reviews and Meta‐Analyses extension for Scoping Reviews (PRISMA‐ScR) [38] have been considered for reporting purposes (Supplementary 2).

### 2.3 Study Selection and Data Extraction

Two investigators independently (D.P. and S.M.) have screened titles and abstracts of all records to identify potentially relevant publications. A third researcher (A.C.) was included in the selection process in case of any disagreements. The inclusion criteria for this review were: studies evaluating the presence of nurse‐led care model on the management of patients with atrial fibrillation undergoing to ablation procedure, in SDD, ≥ 18 years of age, which enrolled more than 10 adults. Studies meeting any of the following exclusion criteria were excluded from the present review: non‐English language, animal studies, meta‐analysis, and review articles.

In this review, SDD was defined according to the terminology commonly used in the literature, indicating discharge occurring on the same calendar day as the ablation procedure, regardless of the exact time of discharge [39].

Two reviewers (D.P. and S.M.) independently performed the data extraction using an Excel sheet to tabulate the following data: first author's name, year of publication, study design, country of origin, purpose of the study, nature and size of the sample, follow‐up period, type of nurse‐led intervention, outcomes measure, same‐day discharge procedure compared with overnight stay procedure, complications, and post‐procedural problems. In terms of outcome measures, the objective was to extract data on postoperative outcomes that can evaluate the effectiveness of interventions for patients with atrial fibrillation. Such outcome measures may encompass, but were not restricted to, cardiovascular hospitalization and cardiovascular death. Data were also collected on the differences in terms of enhanced performance between the same‐day discharge procedure and the overnight stay (ONS) procedure in terms of thromboembolic events or cardiac events. These data contributed to assessing the impact of the procedure, including nurse‐led health management and nursing interventions, on reducing complications and enhancing patient safety. Data concerning complications and symptoms experienced by postoperative atrial fibrillation patients, such as surgical complications, palpitations, shortness of breath, fatigue, or chest discomfort, were extracted. This information aided in evaluating the effectiveness of nurse‐led interventions in managing and alleviating symptoms.

## 3 Results

### 3.1 Study Selection

**FIGURE 1:**
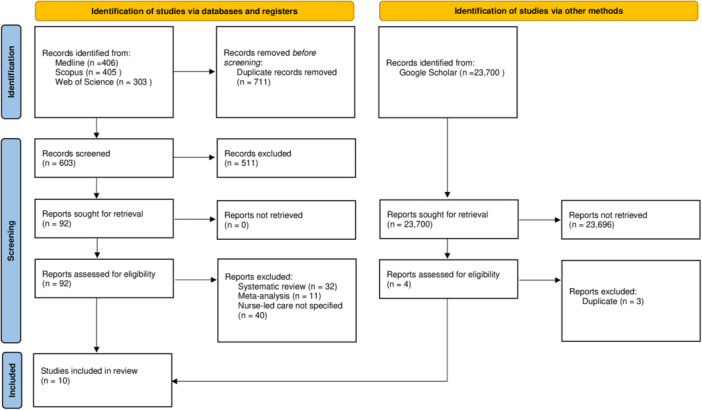
PRISMA 2020 flow diagram for new systematic reviews which included searches of databases, registers, and other sources. *From:* Page MJ, McKenzie JE, Bossuyt PM, Boutron I, Hoffmann TC, Mulrow CD, et al. The PRISMA 2020 statement: an updated guideline for reporting systematic reviews. BMJ 2021;372:n71. https://doi.org/10.1136/bmj.n71. For more information, visit: http://www.prisma-statement.org/.

A total of 25,014 studies were retrieved and 23,792 unique results remained for the initial title and abstract screening. Results were screened and 96 manuscripts underwent full‐text review. Finally, only 10 articles met full inclusion criteria (Figure 1). Overall, 4776 patients were enrolled in the 10 studies (range: 41–2418), five of which enrolled fewer than 250 patients [20, 40, 41, 42, 43]. Studies included two multicenter cohort studies, four retrospective studies, three observational studies, and one real‐world cohort analysis (Table 1). All studies were conducted in Europe [20, 40, 41, 44, 45, 46] and North America [21, 42, 43, 47], and they presented a study period that collectively extended from 2010 to 2023. A summary of the characteristics and purposes of each study is reported in Table 1.

**TABLE 1:** Design and characteristics of the included studies.

References	Country	Inclusion period	Design	Purpose of the study	Nature and size of the sample
Bartoletti, 2019	UK	2014–2017	RS	To investigate the safety and feasibility of early mobilization and SDD following streamlined peri‐ablation management for AF	169 SDD and 642 ONS AF ablation cases included
Ashwin Reddy, 2020	UK	2017–2018	RS	To assess the overall effectiveness of our day case service, in particular identification of operational or procedural barriers to SDD, complication rates and long‐term outcomes	128 SDD and 283 ONS AF ablation cases included
Deyell, 2020	Canada	2010–2014	MCS	To evaluate the efficacy, health care utilization, and safety of an SDD protocol.	2418 SDD and 283 ONS AF ablation cases included
Escobar‐Cervantes, 2019	Spain	2013–2018	OBS	To assess the safety of early hospital discharge (< 24 h) after AF ablation	103 SDD cases included
Deyell, 2022	Canada	2018–2019	MCS	To examine the safety, efficacy, and subsequent healthcare utilization of a same‐day discharge protocol for AF ablation	421 SDD cases included
He, 2020	UK	2011–2019	RS	To evaluate the safety and cost‐effectiveness of same‐day complex left‐atrial arrhythmia ablation.	414 SDD and 553 ONS AF ablation cases included
Creta, 2020	UK	2016–2019	RS	To investigate the safety and cost‐effectiveness of SDD	727 SDD and 1901 ONS AF ablation cases included
Rajendra, 2020	US	2017–2018	RCA	To report on the feasibility and safety of an SDD protocol	41 SDD and 41 ONS AF ablation cases included
Forman, 2023	Canada	2022	OBS	To assess the feasibility of implementing a nurse‐led phone call on day‐1 after AF ablation and impact on 30‐day readmission rates, ER visits and patient‐reported satisfaction.	50 SDD cases included
Espinosa, 2024	Spain	2021–2023	OBS	To implement a streamlined, nurse‐coordinated SDD program following a standardized protocol.	331 SDD cases included

Abbreviations: AF, atrial fibrillation; MCS, multicenter cohort study; NA, not assessed; OBS, observational study; ONS, overnight stay procedure; RCA, real‐world cohort analysis; RS, retrospective study; SDD, same‐day discharge.

### 3.2 Nurse‐Led Intervention

The nurse‐led interventions identified in the methodologies of the included articles were varied and comprehensive. Bartoletti et al. [40], provided a dedicated heart rhythm nurse hotline accessible during working hours, which allowed for differentiation between non‐complication‐related symptoms and potential complications, enabling early intervention when necessary. In the work of Rajendra et al. [42], all patients were called by the nursing team the next morning of the operation and 70 days after discharge from the hospital to check for complications. In the methodology adopted by authors Forman et al. [43], patients received, from the nursing team, a phone call on day 1 postoperatively to assess post‐procedure complications and reinforce the education received by nurses regarding the detection of clinical symptomatology referable to complications. In the approach described by Ashwin Reddy et al. [20], discharge could be nurse‐led if pre‐authorized by the clinician in the post‐procedure plan. In this way, the nursing team could independently discharge a patient who was free of complications or who did not require further clinician visits. In the 2020s work of Deyell et al. [47], the decision to admit a patient for overnight monitoring was left not only to the discretion of the attending physician but also to the nursing team. In addition, all discharged patients were followed by interdisciplinary atrial fibrillation clinics with telephone nursing visits at 7–10 days and in‐person visits at 3, 6, and 12 months. Finally, all patients had telephone access to atrial fibrillation clinic nurses. The study by Escobar‐Cervantes et al. [41], was included in our review because discharge from the hospital scheduled < 24 h after admission, was performed by the nursing staff without prior medical examination, unless accidents occurred. In the 2022 work of the group of Deyell et al. [21], was again shared between the attending physician and the nursing team, with telehealth support provided by nursing staff. However, in the work done by He et al. [44], the nursing staff provided information on self‐care once home, returning to work, driving, flying, what to expect and who to contact if needed. Creta et al. [45], outlined a protocol where, in the absence of clinical concerns, nurses discharged eligible patients after a clinical assessment using a standardized workflow, including femoral access site evaluation, observations, and completion of evaluation forms. Finally, the study by Espinosa et al. [46], employed a standardized protocol through which an SDD nurse coordinator, that is, a nurse specializing in outpatient cardiac interventions, was established and responsible for the entire SDD protocol, including patient selection, patient flow, hospital logistics, patient and family education, discharge, and short‐term follow‐up.

### 3.3 Same‐Day Discharge Compared With Overnight Stay Procedure

Among the 10 papers included, only six compared procedures performed with same‐day discharge to procedures with planned overnight hospitalization. In the work of Bartoletti et al. [40], they point out the following as improving aspects of the procedure performed in same‐day discharge, compared to the classical one involving overnight hospitalization: shorter procedure, performed under sedation, less linear injury and cardio electric version, less economic costs. While Ashwin Reddy et al. [20], note the following aspects: shorter, more likely to start in the morning and less likely to require general anesthesia, less distance for the patient to travel, less economic cost. The 2020s work of Deyell et al. [47], observes improvements with the same‐day discharge method with respect to the following parameters: no association with higher rates of hospital readmission or complications after same‐day discharge. While He et al. [44] indicate the following aspects as elements of improvement given by the same‐day discharge procedure: conscious sedation, cost‐effective. Finally, the comparison between same‐day discharge and hospitalization during the night of the authors Creta et al. and Rajendra et al. [42, 45], determines the first procedure as an improvement for the best cost‐effectiveness.

### 3.4 Outcomes (% Complication Rate)

The overall complication rate following treatment in SDD ranged from 0% to 18% as shown in Table 2. Analyzing the frequency of complications out of the total number of patients evaluated in the included studies, we can mention the following: only one study indicated 0% complications following procedure in SDD mode [42]. Ashwin Reddy et al. [20] reported ran overall complication rate of 2.3%, whereas in Deyell et al.'s 2020 study [47], complication rates from same‐day discharge to 30 days were 0.37%. The complication rate was 1.0% in three studies [21, 41, 44] and 1.6% at 30 days in another two studies [45, 46]. In the work of Bartoletti et al. [40] elaborate on the data by representing it as follows: complications occurred at a rate of 0.7% during procedure, among day cases 3.5% had minor problems, and 2.1% needed re‐hospitalization after discharge. The unplanned overnight hospital admissions were due to minor complications (7.3%) and 0% of major post‐discharge complications in the study of Espinosa et al [46]. Finally, the study by Forman et al. [43] had the highest rate of post‐surgical complications, at 18%.

**TABLE 2:** Patients' outcomes rates and nurse‐ or physician‐led care models.

Author	Total SSD patients	Follow‐up period	Nurse‐led intervention	Outcomes (% complication rate)	SDD compared with ONS	Type of complications
Bartoletti, 2019	143	NA	Telephonic number for dedicated heart rhythm nurse helpline, accessible at any time during working hours	During the procedure 0.7% cases, among day‐cases 3.5% experienced minor problems, 2.1% needed re‐hospitalization post‐discharge	Shorter procedure, performed under sedation, less linear lesions and electrical cardio version, less economic cost	Transient right phrenic nerve palsy, symptomatic vasovagal events, bleeding, nausea and vomiting, pericarditis chest pain
Ashwin Reddy, 2020	128	3–6 months	Discharge could be nurse‐led if stipulated by the operator in the post‐procedure plan	Overall complication rate was 2.3%	Shorter, more likely to Commence in the morning and less likely to require general anesthetic, less far to travel, less economic cost.	Phrenic nerve palsy, cardiovascular death, migraine
Deyell, 2020	2418	Telephonic visits at 7–10 days and in‐person visits at 3, 6, and 12 months	The decision to admit a patient for overnight monitoring was left to the discretion of the treating physician and nursing team. Patients were followed up by interdisciplinary AF clinics with telephone visits at 7–10 days and in‐person visits at 3, 6, and 12 months. All patients had telephone access to AF clinic nurses.	Complication rates from same‐day discharge to 30 days were 0.37%	Not associated with higher hospital readmission or complication rates after same‐day discharge	Composite outcome, stroke, TIA, embolism, bleeding
Escobar‐Cervantes, 2019	103	NA	Hospital discharge was scheduled < 24 h after admission by nursing staff without prior medical visit unless incidents occurred	Complication rate was 1.0%	NA	Arterial pseudo aneurysm
Deyell, 2022	421	30 days	The decision to admit a patient for overnight monitoring was left to the discretion of the treating physician and nursing team, telehealth support from nurses	Complication rate was 1.0%	NA	Bleeding, effusion
He, 2020	414	20 months	Nurses provided information on ongoing self‐care once at home, returning to work, driving, flying, what to expect and who to contact if required.	Complication rate was 1.0%	Conscious sedation, cost‐effective	Vascular injury, bleeding, pericardial effusion, reversible phrenic nerve palsy, stroke, arrhythmia, heart block requiring pacing, death
Creta, 2020	727	30 days	In the absence of clinical concerns, the nurse discharged suitable patients, clinical assessment was performed by the nurse before discharge using a pre‐specified workflow, including evaluation of the femoral access site, observations, NEWS score.	Complication rate at 30 days was 1.6%	Cost‐effective	Phrenic nerve palsy, stroke, major vascular complication, nausea and vomiting, pericarditis chest pain, femoral complication, AF, chest infection, hemoptysis
Rajendra, 2020	41	70 days	A nurse called all patients the following morning and after 70 days to elicit evidence of complications.	Complication rate was 0%	Cost‐effective	No SDD‐related complications occurred, and no return visits resulted from the follow‐up calls.
Forman, 2023	50	30 days	Patients received a day‐1 phone call to assess for post‐procedure complications and reinforce education	Clinic care‐provider based on nurse assessment was required for 18%	NA	Chest pain, arrhythmias, medication adjustment
Espinosa, 2024	331	Smartphone‐based virtual visits at 1 and 3 days, in‐person visits at 14 days and 3 months, virtual visit at 6 and 12 months	− Pre‐ablation visit (1 week pre‐ablation): Nurse‐led AF outpatient clinic. − Peri‐procedural coordination: Patient/support person education & communication − Early post‐discharge follow‐up: Smartphone‐based virtual visits	The unplanned overnight hospital admissions were due to minor complications (7.3%). Zero percent of major post‐discharge complications. Complication rate at 30 days was 1.6%	NA	Femoral bleeding, TIA
Total patients	4776					

Abbreviations: AF, atrial fibrillation; NA, not assessed; ONS, overnight stay procedure; SDD, same‐day discharge; TIA, transient ischemic attack.

### 3.5 Type of Complications

This review also categorized the types of complications, reported across all included studies. The main complications encountered were as follows:

− Cardiovascular: transient right phrenic nerve palsy, symptomatic vasovagal events, bleeding, pericarditis chest pain, cardiovascular death, arrhythmia, heart block requiring pacing, femoral complication, and atrial fibrillation.
− Gastrointestinal: nausea and vomiting.
− Neurological: migraine, embolism, TIA, effusion, and stroke.
− Surgical: chest infection, hemoptysis, and arterial pseudo aneurysm.

Only one study, by Rejandra et al. [42], reported no complications or return hospitalizations based on follow‐up calls.

## 4 Discussion

This scoping review offers a novel contribution to the literature by specifically examining the role of nurse‐led interventions within SDD pathways after AF ablation—an area underexplored in previous systematic reviews and meta‐analyses. While earlier studies have established the general safety and feasibility of SDD following AF ablation [22, 23], this review uniquely highlights how structured nursing involvement—such as discharge autonomy, follow‐up protocols, and telemonitoring—can enhance patient outcomes and safety within SDD protocols. Moreover, by synthesizing data from real‐world settings, it contextualizes the operational role of nurses in managing complications and ensuring continuity of care post‐ablation. This targeted focus on the care delivery model represents a shift from prior reviews that concentrated primarily on procedural outcomes, offering actionable insights into optimizing interdisciplinary strategies to support SDD implementation. By addressing the organizational dimension of post‐ablation care, this study bridges a critical gap in existing knowledge and provides a foundation for developing standardized, nurse‐led protocols across healthcare systems.

Nurse‐led interventions in the studies varied from dedicated hotlines to follow‐up calls to independent discharge decisions, showing improved early complication detection and patient education. Among the studies, SDD was associated with shorter procedures, reduced costs, and similar or lower complication rates compared to ONS. Complication rates for SDD ranged from 0% to 18%, with most studies reporting low rates (e.g., 0.37% and 1.0%). This interim evaluation highlights the effectiveness and safety of nurse‐led interventions and SDD, with most milestones and deliverables met on time, ensuring continuous improvement and goal alignment. The results of this review are consistent with existing literature on the effectiveness of nurse‐led programs, and research has shown that nurse‐led interventions, in the context of same‐day discharge, are associated with significantly higher efficacy and safety compared to standard care [48, 49]. These findings are also in line with the large meta‐analysis by Zylla et al. [24], which reviewed the most recent evidence on same‐day discharge after atrial fibrillation (AF) ablation, showing that short‐term and 30‐day complications, re‐hospitalizations/ER visits, and 30‐day mortality were rare and not elevated after SDD compared to ONS. This variability in complication rates likely reflects heterogeneity in several key factors, including differences in patient selection (with higher‐risk, older patients or those with multiple comorbidities tending to have higher rates), variability in follow‐up duration and intensity (with longer or more rigorous follow‐up detecting more late or minor events), and differences in ablation techniques, procedural protocols, and nurse‐led intervention structures [44, 46]. In addition, inconsistent definitions and reporting of complications across studies may contribute to the observed range [21, 46]. Another aspect that was not consistently reported across studies is anticoagulation management. Most publications did not clarify whether anticoagulation was continued according to guideline recommendations or whether any temporary dose interruptions were made. Taken together, these elements highlight the need for more standardized clinical pathways, follow‐up strategies, and reporting frameworks to accurately assess the safety profile of SDD after AF ablation.

Nurse‐led discharge involves an early discharge planning program, coordinated with other healthcare professionals, which can take place on the same day, reducing readmission rates and improving continuity of care [50]. This approach has shown benefits in reducing hospital resource utilization [46, 48] and enhancing patients' quality of life [49], especially for those with chronic diseases [51].

Nurse‐managed dedicated telephone lines provide continuous support to patients, allowing them to quickly address doubts and issues. This service has been shown to improve patient satisfaction [52] and reduce post‐SSD complications due to the constant availability of nurses for advice and support [53, 54]. In fact, patient education provided by nurses is crucial for improving self‐management and health awareness [49]. Educational interventions help reduce patient anxiety and build a strong nurse‐patient relationship [55]. However, these interventions often focus mainly on physical health. To further enhance the service, it would be beneficial to adopt more person‐centered decision‐making tools that also address patients' psychosocial needs [53].

Same‐day discharge effectively reduces the use of healthcare resources, such as staff and hospital beds, potentially allowing for an increase in AF ablation procedures in the future. The efficiency and cost‐effectiveness of same‐day discharge following AF ablation have been well‐documented. However, the overall savings in time, personnel, and hospital beds come with certain costs [21, 45, 47, 56]. In addition to structural changes, same‐day discharge necessitates more efficient in‐hospital logistics, coordination, and organizational efforts [46]. The implementation of a dedicated SDD coordinator would be crucial in streamlining the entire same‐day discharge protocol and patient pathway. This role could significantly reduce the workload for both physicians and nurses, enhance communication among all involved parties, and lead to high acceptance of the SDD program [57, 58].

The findings of this study have significant implications across various levels of the healthcare system. At the macro level, governments, ministries, and key stakeholders should focus on developing and promoting policies that support the implementation of nurse‐led interventions and same‐day discharge programs. Establishing a regulatory framework to standardize nurse‐led interventions and SDD protocols is crucial to ensure patient safety and quality care.

However, several gaps in current practices and regulatory standards should be addressed to improve clinical outcomes [59]. Despite the growing adoption of nurse‐led protocols, the absence of standardized frameworks across different clinical settings limits the consistency and replicability of results [59]. Commonly reported barriers to large‐scale implementation include high workload, psychological stress, lack of confidence, and limited resources. Conversely, key enablers such as structured education, strong leadership support, and demonstrable improvements in patient outcomes are essential for successful adoption [59]. Moreover, evidence on the application of nurse‐led chronic disease management models in real‐world settings remains limited, particularly with regard to context‐specific barriers and facilitators [60]. To overcome these challenges, implementation science approaches—such as the Theoretical Domains Framework (TDF) or COM‐B—are recommended to identify behavioral and systemic determinants of protocol adoption and to guide targeted interventions [59].

At the meso level, healthcare organization leaders must commit to integrating nurse‐led interventions and SDD programs into their service offerings. This requires setting clear goals and performance metrics, investing in the necessary infrastructure like dedicated hotlines and telehealth services, and providing continuous education and training for nurses. Ensuring that nurses are equipped with the skills needed for effective discharge planning and post‐discharge support is essential. Healthcare organizations should also promote multidisciplinary coordination to ensure seamless patient transitions from hospital to home, involving regular communication between nurses, physicians, and other healthcare professionals. However, the wider adoption of nurse‐led care faces barriers, such as limited resources, insufficient training opportunities, restrictive regulations, and resistance from traditional physician‐led structures. Implementing these models often requires substantial investment in workforce development and advanced training, while variations in nurses' legal scope of practice can hinder their full application [61, 62]. Cultural and organizational resistance—particularly in physician‐dominated systems—may further limit role expansion. Overcoming these challenges will require targeted policy reform, sustained investment in education, and advocacy to fully leverage nurses' potential in advanced clinical roles [63].

At the micro level, healthcare providers, particularly nurses, should adhere to established SDD protocols and guidelines to provide consistent and high‐quality care. Patient education is a critical component, with nurses focusing on teaching patients about self‐care, symptom management, and when to seek medical help through discharge education sessions and follow‐up calls. For example, nurse‐led interventions could also be leveraged to enhance both short‐ and long‐term adherence to glucagon‐like peptide‐1 receptor agonists (GLP‐1RAs), which have demonstrated remarkable efficacy in maintaining weight control, optimizing metabolic regulation, and reducing recurrence risk after atrial fibrillation ablation [64]. Through structured education, follow‐up consultations, and motivational counseling, trained nurses can address adherence barriers and support sustained clinical benefits post‐ablation.

### 4.1 Strengths and Limitations

Despite the insights gained from this scoping review, several limitations should be acknowledged. First, the small number of articles retrieved and included during the review process. However, the strength of the study lies in its rigorous, reproducible and widely used methodology throughout the literature, which adheres to widely recognized guidelines within the scientific community. Another limitation may have been the inclusion of articles only in English. This choice may have excluded relevant studies in other languages. SDD after atrial fibrillation ablation is a new method of treating atrial fibrillation, and there is research in journals in languages other than English that could have provided more information about the services provided by nurses. In addition, although there are guidelines for the design, interpretation, and writing of various types of systematic reviews [65, 66] structured guidance for scoping reviews—particularly on quality assessment—remains scarce [38]. As scoping reviews typically do not formally assess the methodological quality of included studies, scholars may overlook potential biases, leading to results of uncertain reliability.

Another limitation is that this work only included studies from four developed countries (Canada, the United States, the United Kingdom, and Spain), with over half conducted in Canada and the United Kingdom. Therefore, the results of this review may not be generalizable to health systems in other countries around the world.

Finally, the literature search and databases used in this review may not have captured all studies that examined nurse‐led interventions in the management of SSDs after AF ablation. Notably, this study is the first to review nurse‐led management in the treatment of AF in SDD approach.

### 4.2 Further Issues

Further high‐quality RCT studies are needed in the future to explore the feasibility of implementing a nurse‐led follow‐up call within the first day after atrial fibrillation ablation and its impact on 30‐day readmission rates, emergency department visits, and patient satisfaction. It would be worthwhile to evaluate, comparing the differences between treatment in SSD vs. ONS, the effectiveness, efficiency and feasibility of a dedicated cardiac rhythm nurse hotline, available during working hours or for scheduled hospital discharges without prior physician consultation. These evaluations involve the introduction of a new concept of ambulatory cardiac nurses. This concept requires nurses to have specific training and gain experience in peri‐procedural care of patients undergoing invasive cardiovascular procedures. Before such models can be widely adopted, however, clearer standardization of post‐procedural safety assessments is needed. In this regard, it is noteworthy that none of the included studies reported a same‐day echocardiographic evaluation prior to discharge. This absence represents a methodological gap, as structured imaging could contribute to more uniform and objective safety criteria across centers. Standardization of interventions and outcome measures, inclusion of diverse populations and settings, and evaluation of the economic implications of nurse‐led interventions should be prioritized.

## 5 Conclusion

The main findings of this scoping review are that nurse‐led interventions have the potential to have a significant impact on the care of patients in same‐day discharge after atrial fibrillation ablation by providing comprehensive management, education, and support; nurses can improve patient outcomes, enhance quality of life, and reduce health care burdens for both patients and providers.

## Author Contributions

**Domenico Pascucci:** conceptualization, data curation, formal analysis, investigation, methodology, software, writing – original draft, writing – review and editing. **Silvia Martinelli:** conceptualization, data curation, formal analysis, investigation, methodology, software, writing – original draft, writing – review and editing. **Mario Cesare Nurchis:** conceptualization, supervision, validation, writing – review and editing. **Antonello Cocchieri:** conceptualization, supervision, validation, writing – review and editing. **Francesca Notarangelo:** supervision, validation, writing – review and editing. **Maria Lucia Narducci:** supervision, validation, writing – review and editing. **Walter Ricciardi:** supervision, visualization, writing – review and editing. **Gianfranco Damiani:** supervision, visualization, writing – review and editing.

## Acknowledgments

**The Nursing and Public Health Group**

**Elena Cristofori**, Università Cattolica del Sacro Cuore, Rome, Italy; **Vittorio De Vita**, Università Cattolica del Sacro Cuore, Rome, Italy; **Anna Nisticò**, Università Cattolica del Sacro Cuore, Rome, Italy; **Lia Olivo**, Università Cattolica del Sacro Cuore, Rome, Italy; **Manuele Cesare**, Center of Excellence for Nursing Scholarship, Rome, Italy; **Erasmo Magliozzi**, University of Rome Tor Vergata, Rome, Italy.

## Funding

The authors received no specific funding for this work.

## Disclosure

The lead author Domenico Pascucci affirms that this manuscript is an honest, accurate, and transparent account of the study being reported; that no important aspects of the study have been omitted; and that any discrepancies from the study as planned (and, if relevant, registered) have been explained.

## Ethics Statement

This study is a review of current evidence and therefore did not require study specific ethical approval.

## Conflicts of Interest

The authors declare no conflicts of interest.

## Supporting information

Supplementary 1.

Supplementary 2.

## Data Availability

Data sharing is not applicable to this article, as no new data were created or analyzed in this study.
