# Supplementary material for: Same‐Day Discharge After Atrial Fibrillation Ablation. A Scoping Review of Nurse‐Led Care Model
Source: Health Sci Rep. 2026 Mar 19;9(3):e72127. doi: 10.1002/hsr2.72127 (PMC13097602; doi:10.1002/hsr2.72127)
Supplement: Supplementary file 1 — Supplementary 1. [file HSR2-9-e72127-s001.docx]

Search string

| (“Early discharge” OR “Same-Day procedure” OR “Same day procedure” OR “Same-Day dismissal” OR “Same day dismissal” OR “Same Day Discharge” OR “Same-day discharge” OR “SDD” OR “Outpatient ablation” OR “one day” OR “Day Hospital” OR “Day Surgery” OR “Day Service”) AND (“AF” OR “Atrial fibrillation” OR (“ablation” AND (“AF” OR “Atrial Fibrillation”))) |
| --- |
